# Implementation of five machine learning methods to predict the 52-week blood glucose level in patients with type 2 diabetes

**DOI:** 10.3389/fendo.2022.1061507

**Published:** 2023-01-20

**Authors:** Xiaomin Fu, Yuhan Wang, Ryan S. Cates, Nan Li, Jing Liu, Dianshan Ke, Jinghua Liu, Hongzhou Liu, Shuangtong Yan

**Affiliations:** ^1^ Department of Endocrinology, The First Medical Center, Chinese PLA General Hospital, Beijing, China; ^2^ Department of Emergency Medicine Stanford Healthcare TriValley, Stanford University School of Medicine, Stanford, Pleasanton, CA, United States; ^3^ Department of Endocrinology, The Second Medical Center & National Clinical Research Center for Geriatric Diseases, Chinese PLA General Hospital, Beijing, China; ^4^ Clinics of Cadre, Department of Outpatient, The First Medical Center, Chinese PLA General Hospital, Beijing, China; ^5^ Department of Orthopedics, Fujian Provincial Hospital, Fuzhou, China; ^6^ Beijing Tongren Eye Center, Beijing Tongren Hospital, Capital Medical University, Beijing, China

**Keywords:** type 2 diabetes, machine learning, algorithms, blood glucose, prediction model

## Abstract

**Objective:**

For the patients who are suffering from type 2 diabetes, blood glucose level could be affected by multiple factors. An accurate estimation of the trajectory of blood glucose is crucial in clinical decision making. Frequent glucose measurement serves as a good source of data to train machine learning models for prediction purposes. This study aimed at using machine learning methods to predict blood glucose for type 2 diabetic patients. We investigated various parameters influencing blood glucose, as well as determined the most effective machine learning algorithm in predicting blood glucose.

**Patients and methods:**

273 patients were recruited in this research. Several parameters such as age, diet, family history, BMI, alcohol intake, smoking status et al were analyzed. Patients who had glycosylated hemoglobin less than 6.5% after 52 weeks were considered as having achieved glycemic control and the rest as not achieving it. Five machine learning methods (KNN algorithm, logistic regression algorithm, random forest algorithm, support vector machine, and XGBoost algorithm) were compared to evaluate their performances in prediction accuracy. R 3.6.3 and Python 3.12 were used in data analysis.

**Results:**

The statistical variables for which p< 0.05 was obtained were BMI, pulse, Na, Cl, AKP. Compared with the other four algorithms, XGBoost algorithm has the highest accuracy (Accuracy=99.54% in training set and 78.18% in testing set) and AUC values (1.0 in training set and 0.68 in testing set), thus it is recommended to be used for prediction in clinical practice.

**Conclusion:**

When it comes to future blood glucose level prediction using machine learning methods, XGBoost algorithm scores the highest in effectiveness. This algorithm could be applied to assist clinical decision making, as well as guide the lifestyle of diabetic patients, in pursuit of minimizing risks of hyperglycemic or hypoglycemic events.

## 1 Introduction

Diabetes counts as one of the leading chronic diseases that have significant impacts on people worldwide. According to a report issued by the International Diabetes Federation (IDF), in 2021, diabetic patients have reached 536.6 million and the prevalence is estimated to exceed 783.2 million by 2045 in the age group of 20 to 79 years old (1). More than 90% of all patients suffer from type 2 diabetes. In China, it is projected that 145 million people are diabetic (2),while in the US, the number is 34.2 million (3).

Certain tests such as fasting plasma glucose, 2h-PG, and the level of HbA1c are considered as appropriate diagnostic criteria (4). The American Diabetes Association suggests the use of validated tools to identify and screen affected adults to assess the risk factors leading to the onset of diabetes mellitus (5).Main pathological defects in patients suffering from type 2 diabetes include insulin resistance, as well as impaired insulin secretion due to malfunctioning of pancreatic β cells. In addition, five other pathophysiological conditions contribute to glucose intolerance in diabetic patients. These include: lipo-toxicity, higher glucagon production by α-cells, enhanced hepatic sensitivity to glucagon, increased glucose reabsorption through glucose transporter-2 by the kidneys, and CNS resistance to the suppressive effects of insulin leading to appetite dysregulation and abnormal weight gain. All of these factors lead to the maintenance of high glucose levels in the blood. Other factors aggravating type 2 diabetes include glucotoxicity, inflammation, and oxidative stress. Inflammation has been reported to alter the concentration of certain cytokines and chemokines, alter the number and activation status of leukocytes and facilitate tissue fibrosis and leukocyte apoptosis, and thus is crucial in the pathophysiology of type 2 diabetes (6–9).

Symptoms of diabetes include dehydration, blurry vision, sudden weight loss, polyuria, polydipsia, and polyphagia. Diabetic patients are more susceptible to heart, brain, and vascular diseases. Diseases of the cardiovascular system contribute to the majority of mortalities in diabetic patients (10). Therefore, paying due attention to blood glucose levels in patients suffering from diabetes is critical. Regular monitoring as well as assessment are important both to maintain appropriate blood glucose levels in these patients and to avoid unnecessary short and long-term complications. The normal blood glucose levels vary depending on various aspects including physical activity, and 70-180 mg/dl is considered a safe range to avoid any sudden or gradual complications (11). Regulation and maintenance of optimum glucose levels in the blood are crucial for the quality of life. The better the regulation, the less the chances will be of chronic complications of diabetes. It is important to prevent both hypo and hyper-glycemic conditions for effective diabetes management. The blood glucose concentration is affected by multiple factors, and it is ideal to use the historic values as predictive inputs (3, 12).

Proper diabetes management requires consideration of various factors including tailored food intake, medication, insulin levels, and physical activity, in the hope to achieve precision control for each patient. Presently, oral drugs and insulin injections are commonly used to treat diabetes (13). Early management of risk factors and proper intervention are crucial (12). This study was conducted with the aim of supporting patients with medical or lifestyle decisions (for example, meal planning, proper insulin dosages, or consuming certain nutrients) by predicting their blood glucose levels using machine learning algorithms.

Machine learning methods have played supportive parts in building appropriate models by learning and assessing patterns through data. Such models help discover underlying correlations and future projections from the data. Typically, the features are engineered with prior knowledge, as well as statistical analysis (mean, standard deviation, PCA et al). The algorithms’ performances such as logistic regression and k-nearest neighbors depend on the given data (14).

In the present research, we first conducted a training experiment over a set of data from the patient cohort, then performed the evaluation experiment in a test dataset. Five different algorithms were compared.

## 2 Patients and methods

### 2.1 Object of study

This is a retrospective study. Baseline survey and follow-up information of multicenter type 2 diabetes patients were collected. The survey period of the database was from October 2016 to December 2017. Elderly patients with type 2 diabetes aged 60 years and above were randomly selected from 150 provincial hospitals in 21 provinces across China. A total of 2652 cases were included, 1396 were males and 1256 were females. The age range was 65-77 years, with a median age of 69 years. Out of those patients in the database, only 273 patients had complete data of blood glucose levels and other parameters at 16-week and 52- week follow-up. 273 patients were enrolled. Medical Ethics Committee of the Chinese PLA General Hospital gave approval to this research (No. S2015-038-01). Outcome categorical variable was applied to establish prediction models using algorithms of random forest, support vector machine, logistic regression, KNN, and XGBoost. The ROC curve, accuracy, precision, recall, F1 and other indicators were utilized to compare the prediction effect.

### 2.2 Composition of candidate indicators

In accordance with the Chinese guideline for type 2 diabetes (15), patients with glycated hemoglobin less than 6.5% after 52 weeks were considered to have achieved glycemic control, and the rest were considered to have not succeeded. Based on the survey data, follow-up data, and previous studies, age, gender, experimental grouping, family history, education level, dietary assessment, complications(retinopathy, kidney disease, peripheral neuropathy, peripheral atherosclerosis, intermittent claudication), hypertension, drinking status, smoking status, BMI, pulse rate, and key biochemical indicators(HDL, Hb, K, Na, Cl, CO_2_, Ca, P, AKP, GPT, GOT, rGT) were considered as candidate indicators. We built machine learning models on the statistically significant variables obtained from univariate analysis as predictor variables.

### 2.3 Data pre-processing

Missing values of candidate variables were filled using median for continuous variables and mode for categorical variables. Stratified sampling was performed based on glycemic control effects. The allocation of the training set vs the testing set was 80%: 20%.

### 2.4 Statistical methods

R 3.6.3 software was used to perform statistical analysis. Comparisons between groups of continuous variables were calculated through t-test or ANOVA, and continuous variables that were not normally distributed were analyzed using rank sum test. Machine learning was performed using Python 3.12 software. p< 0.05 was considered statistically significant.

### 2.5 Forecasting models

#### 2.5.1 Logistic regression prediction model

In machine learning, the method of logistic regression is frequently used. It is a supervised classification model with considerable simplicity and the performance of this algorithm is superb. Patients with substandard glycemic control were used as the case group and those with standard glycemic control were used as the control group (16). The equation of logistic regression is:


y=e^(b0+b1*x)/(1+e^(b0+b1*x))

#### 2.5.2 Random forest model

Random forest model is an integrated learning method, which introduces randomly attributed selection based on decision trees. It is a classification algorithm that uses multiple weak classifiers combined into one strong classifier. It is extensively utilized in the research of classifying and predicting because of its simplicity, easy implementation, low computational overhead, high adaptability to data, and ability to handle large datasets (17).

#### 2.5.3 Support vector machine models

A support vector machine is a class of generalized classifier. It is a robust linear supervised learning and it performs binary classification. In this algorithm, instead of using probability models, hyperplanes aimed for classification and regression are built. The decision boundary is a maximum-margin hyperplane (18).

#### 2.5.4 KNN model

The k-nearest neighbor method is based on regression model. The k-nearest neighbor assumes that given a training dataset in which the class of instances has been determined, classification is done by predicting new instances based on the class of their k nearest neighbor training instances, for example, by majority voting (19).

#### 2.5.5 XGboost algorithm

XGBoost is a competent algorithm of gradient boosting decision tree. It incorporates improvements based on the original GBDT algorithm, making the model much more effective. The fundamental element of this algorithm is the application of integration. Multiple weak learners merge into one strong learner through mathematical methods. The final result is accomplished through integration of the results of multiple trees, so as to achieve the improvement of the whole model effect (20).

## 3 Results

### 3.1 Analysis of baseline information

In total, 273 cases were studied in the statistical analysis. **Table 1** illustrated the basic distribution of each parameter among patients. 119 patients were female, 154 patients were male.

**Table 1:** Baseline characteristics data.

Variables	Cases (n = 273)
HbA1c(%), n (%)	
no	225 (82.4)
yes	48 (17.6)
gender, n (%)	
female	119 (43.6)
male	154 (56.4)
age, n (%)	
<75	134 (49.1)
>=75	139 (50.9)
Experiment_Type, n (%)	
Experimental_group_1	67 (24.5)
Experimental_group_2	40 (14.7)
Experimental_group_3	32 (11.7)
baseline	83 (30.4)
regular_group	51 (18.7)
educational_level, n (%)	
Junior_and_below	134 (49.1)
high_and_college	139 (50.9)
BMI, n (%)	
<25	110 (40.3)
>=25	163 (59.7)
pulse, n (%)	
<75	125 (45.8)
>=75	148 (54.2)
smoke, n (%)	
no	235 (86.1)
yes	38 (13.9)
drink, n (%)	
no	198 (72.5)
yes	75 (27.5)
family_history, n (%)	
no	185 (67.8)
yes	88 (32.2)
diet_assessment, n (%)	
good	66 (24.2)
ordinary	207 (75.8)
Retinopathy, n (%)	
no	226 (82.8)
yes	47 (17.2)
Kidney_disease, n (%)	
no	219 (80.2)
yes	54 (19.8)
peripheral_neuropathy, n (%)	
no	176 (64.5)
yes	97 (35.5)
peripheral_atherosclerosis, n (%)	
no	171 (62.6)
yes	102 (37.4)
Intermittent_claudication, n (%)	
no	262 (96.0)
yes	11 (4.0)
hypertension, n (%)	
no	92 (33.7)
yes	181 (66.3)
HDL_C(mmol/L), mean (SD)	1.3 (0.4)
Hb(g/L), mean (SD)	132.5 (14.9)
K(mmol/L), mean (SD)	4.1 (0.4)
Na(mmol/L), mean (SD)	137.9 (15.5)
Cl(mmol/L), mean (SD)	102.8 (9.9)
CO2CP(mmol/L), mean (SD)	25.1 (2.8)
Ca(mmol/L), mean (SD)	2.3 (0.1)
P(mmol/L), mean (SD)	1.1 (0.2)
AKP(U/L), mean (SD)	76.0 (23.0)
GPT(U/L), mean (SD)	25.2 (15.4)
GOT(U/L), mean (SD)	22.6 (10.7)
rGT(U/L), mean (SD)	30.1 (25.3)

50.9% of the patients were equal to or over 75 years old. 59.7% of all patients were overweight. 17.6% of the patients achieved glycemic control, 82.4% didn’t.

### 3.2 Analysis of blood glucose control compliance and non-compliance

Patients were considered to have glycosylated hemoglobin less than 6.5% after 52 weeks as having achieved glycemic control and the rest as not achieving it. The statistical variables for which p< 0.05 was obtained include BMI, pulse, Na, Cl, and AKP (**Table 2**).

**Table 2:** Comparison of baseline characteristics based on HbA1c (%).

Variable	no	yes	P-Value	Test
n	225	48		
HbA1c(%), n (%)				
no	225 (100.0)		<0.001	Chi-squared
yes		48 (100.0)		
gender, n (%)				
female	96 (42.7)	23 (47.9)	0.613	Chi-squared
male	129 (57.3)	25 (52.1)		
age, n (%)				
<75	111 (49.3)	23 (47.9)	0.985	Chi-squared
>=75	114 (50.7)	25 (52.1)		
Experiment_Type, n (%)				
Experimental_group_1	57 (25.3)	10 (20.8)	0.948	Chi-squared
Experimental_group_2	32 (14.2)	8 (16.7)		
Experimental_group_3	27 (12.0)	5 (10.4)		
baseline	68 (30.2)	15 (31.2)		
regular_group	41 (18.2)	10 (20.8)		
educational_level, n (%)				
Junior_and_below	115 (51.1)	19 (39.6)	0.197	Chi-squared
high_and_college	110 (48.9)	29 (60.4)		
BMI, n (%)				
<25	82 (36.4)	28 (58.3)	0.008	Chi-squared
>=25	143 (63.6)	20 (41.7)		
pulse, n (%)				
<75	93 (41.3)	32 (66.7)	0.002	Chi-squared
>=75	132 (58.7)	16 (33.3)		
smoke, n (%)				
no	191 (84.9)	44 (91.7)	0.316	Chi-squared
yes	34 (15.1)	4 (8.3)		
drink, n (%)				
no	162 (72.0)	36 (75.0)	0.807	Chi-squared
yes	63 (28.0)	12 (25.0)		
family_history, n (%)				
no	149 (66.2)	36 (75.0)	0.312	Chi-squared
yes	76 (33.8)	12 (25.0)		
diet_assessment, n (%)				
good	51 (22.7)	15 (31.2)	0.282	Chi-squared
ordinary	174 (77.3)	33 (68.8)		
Retinopathy, n (%)				
no	185 (82.2)	41 (85.4)	0.748	Chi-squared
yes	40 (17.8)	7 (14.6)		
Kidney_disease, n (%)				
no	180 (80.0)	39 (81.2)	1.000	Chi-squared
yes	45 (20.0)	9 (18.8)		
peripheral_neuropathy, n (%)				
no	141 (62.7)	35 (72.9)	0.238	Chi-squared
yes	84 (37.3)	13 (27.1)		
peripheral_atherosclerosis, n (%)				
no	141 (62.7)	30 (62.5)	1.000	Chi-squared
yes	84 (37.3)	18 (37.5)		
Intermittent_claudication, n (%)				
no	215 (95.6)	47 (97.9)	0.695	Fisher's exact
yes	10 (4.4)	1 (2.1)		
hypertension, n (%)				
no	77 (34.2)	15 (31.2)	0.820	Chi-squared
yes	148 (65.8)	33 (68.8)		
HDL_C(mmol/L), median [Q1,Q3]	1.2 [1.0,1.4]	1.2 [1.1,1.5]	0.200	Kruskal-Wallis
Hb(g/L), median [Q1,Q3]	132.0 [125.0,139.4]	132.8 [124.9,139.2]	0.958	Kruskal-Wallis
K(mmol/L), median [Q1,Q3]	4.1 [3.8,4.3]	4.2 [3.9,4.3]	0.353	Kruskal-Wallis
Na(mmol/L), median [Q1,Q3]	139.5 [137.9,141.8]	141.0 [139.0,142.0]	0.009	Kruskal-Wallis

### 3.3 Machine learning approach

#### 3.3.1 Results for the training set of machine learning models

Results of the training set were shown in **Table 3**:

1) KNN (accuracy=0.8486, precision=0.9972, recall=0.6073, F1=0.6811, AUC_PR=0.6197, AUC_ROC=0.8702)
2) Logistic Regression (accuracy=0.6376, precision=0.5693, recall=0.6145, F1=0.5910, AUC_PR=0.3864, AUC_ROC=0.7025)
3) Random Forest (accuracy=0.8119, precision=0.7027, recall=0.7719, F1=0.7357, AUC_PR=0.6621, AUC_ROC=0.8854)
4) Support Vector Machine (accuracy=0.6789, precision=0.5829, recall=0.6291, F1=0.6051, AUC_PR=0.4513, AUC_ROC=0.7480)
5) XGBoost (accuracy=0.9954, precision=0.9972, recall=0.9868, F1=0.9920, AUC_PR=0.9993, AUC_ROC=0.9999)

**Table 3:** Results of the training sets using five machine learning algorithms.

model	accuracy	precision	recall	f1	auc_pr	auc_roc
**Logistic regression**	0.6376	0.5693	0.6145	0.5910	0.3864	0.7025
**Random forest**	0.8119	0.7027	0.7719	0.7357	0.6621	0.8854
**Support vector machine**	0.6789	0.5829	0.6291	0.6051	0.4513	0.7480
**KNN**	0.8486	0.7754	0.6073	0.6811	0.6197	0.8702
**XGBoost**	0.9954	0.9972	0.9868	0.9920	0.9993	0.9999

XGBoost has the highest accuracy and AUC values.

#### 3.3.2 Results for the machine learning model testing set

Results of the testing set were listed in **Table 4**:

1) KNN (accuracy=0.7818, precision=0.5850, recall=0.5556, F1=0.5699, AUC_PR=0.2594, AUC_ROC=0.4867)
2) Logistic Regression (accuracy=0.6727, precision=0.5713, recall=0.6056, F1=0.5879, AUC_PR=0.2947, AUC_ROC=0.6311)
3) Random Forest (accuracy=0.6909, precision=0.5583, recall=0.5778, F1=0.5679, AUC_PR=0.3208, AUC_ROC=0.6311)
4) Support Vector Machine (accuracy=0.6545, precision=0.5621, recall=0.5944, F1=0.5778, AUC_PR=0.2766, AUC_ROC=0.6644)
5) XGBoost (accuracy=0.7818, precision=0.5850, recall=0.5556, F1=0.5699, AUC_PR=0.2924, AUC_ROC=0.6800)

**Table 4:** Results of the testing sets using five machine learning algorithms.

model	accuracy	precision	recall	f1	auc_pr	auc_roc
**Logistic regression**	0.6727	0.5713	0.6056	0.5879	0.2947	0.6311
**Random forest**	0.6909	0.5583	0.5778	0.5679	0.3208	0.6311
**Support vector machine**	0.6545	0.5621	0.5944	0.5778	0.2766	0.6644
**KNN**	0.7818	0.5850	0.5556	0.5699	0.2594	0.4867
**XGBoost**	0.7818	0.5850	0.5556	0.5699	0.2924	0.6800

XGBoost had the highest accuracy and AUC values.

#### 3.3.3 Confusion matrix for different machine learning models

1) KNN

**Figure 1:**
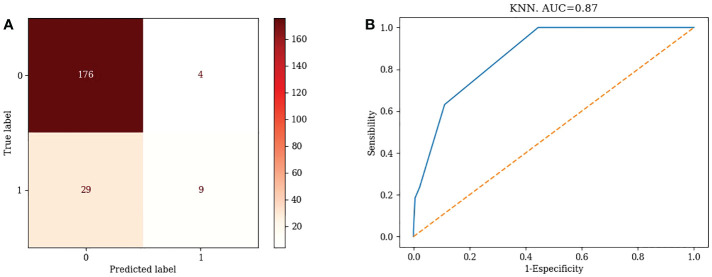
Confusion matrix and ROC curve of KNN algorithm.

TN=176, FP=4, FN=29, TP=9. Accuracy= 84.86%, Misclassification rate=15.14% (**Figure 1A**)

2) Logistic regression

**Figure 2:**
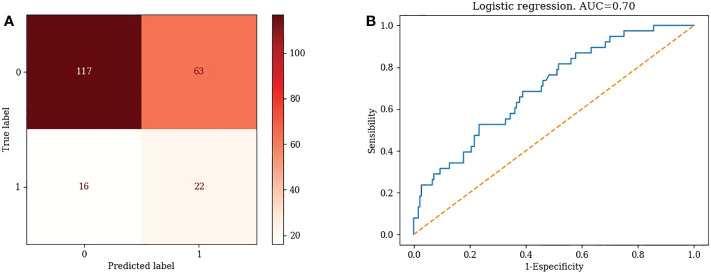
Confusion matrix and ROC curve of Logistic Regression algorithm.

TN=117, FP=63, FN=16, TP=22. Accuracy= 63.76%, Misclassification rate=36.24% (**Figure 2A**)

3) Random Forest Model

**Figure 3:**
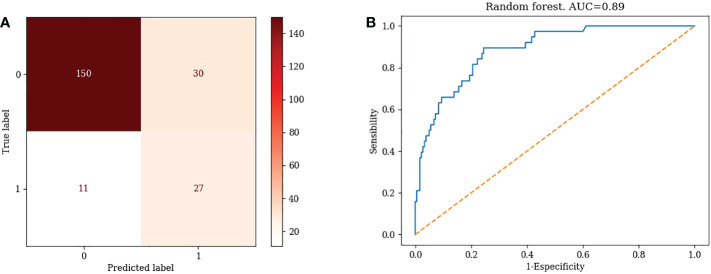
Confusion matrix and ROC curve of Random Forest algorithm.

TN=150, FP=30, FN=11, TP=27, Accuracy=81.19% Misclassification rate=18.81% (**Figure 3A**)

4) Support vector machines

**Figure 4:**
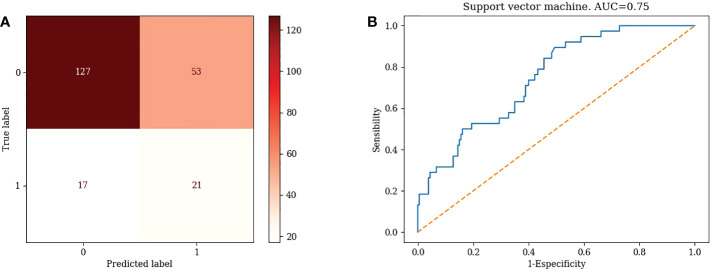
Confusion matrix and ROC curve of Support Vector Machine algorithm.

TN=127, FP=53, FN=17, TP=21, Accuracy= 67.89 %, Misclassification rate= 32.11% (**Figure 4A**)

5) XGBoost

**Figure 5:**
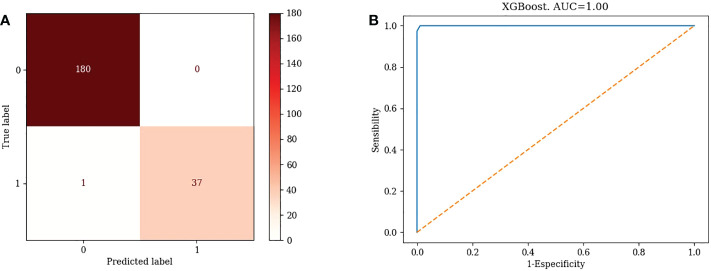
Confusion matrix and ROC curve of XGBoost algorithm.

TN=180, FP=0, FN=1, TP=37. Accuracy=99.54%, Misclassification rate=0.46% (**Figure 5A**)

#### 3.3.4 ROC curves for different machine learning models

1) KNN: AUC=0.87 (**Figure 1B**)
2) Logistic Regression: AUC=0.70 (**Figure 2B**)
3) Random Forest: AUC=0.89 (**Figure 3B**)
4) Support Vector Machine: AUC=0.75 (**Figure 4B**)
5) XGBoost: AUC=1.00 (**Figure 5B**)

XGBoost had the highest AUC value, indicating that XGBoost’s is a good choice for clinical application.

## 4 Discussions

In the occasion of long term non-controlled type 2 diabetes, various micro and macrovascular complications could occur. Microvascular complications arise due to hyperglycemic conditions facilitated by the activation of several pathological mechanisms such as enhanced polyol pathway flux, increased concentration of end products from advanced glycation, expression from AGE receptors, hexosamine flux, activation of proteins kinases, and an increased generation of reactive oxygen and nitrogen. Some of the major microvascular complications include neuropathy, retinopathy, and nephropathy (21, 22). The pathways contributing towards microvascular complications align with macrovascular complications as well. Diabetes is an important risk factor in patients suffering from cardiovascular diseases. Diabetic patients have high mortality rates and tend to be hospitalized for longer periods (23). The socio-economic losses and medical expenses resulting from the treatment of acute or chronic complications for such patients are enormous. It is, therefore, necessary to closely monitor the blood glucose of type 2 diabetes patients. Standard approaches for effective blood glucose management include finger prick testing throughout the day at regular intervals, self-monitoring and recording of blood glucose values, and effective use of glucose monitoring devices. Recent developments in technology have allowed patients to use glucose monitoring sensors, assess glucose levels in subcutaneous space, providing insights into their blood glucose levels. To lessen the workload of healthcare specialists, who are very few as compared to the number of patients, and eliminate the bias factor for decision-making, machine learning and artificial intelligence can be used to facilitate medical practitioners. Deep insights into glucose fluctuations can assist patients to take necessary actions prior to hyper or hypoglycemic events and minimize the occurrence of adverse glycemic conditions.

In our research, parameters for which p< 0.05 was obtained include BMI, pulse, Na, Cl, and AKP. Overweight and obesity are rapidly growing public health problems in recent years, leading to many metabolic risks including type 2 diabetes (24). People with BMI>30 kg/m^2^ are classified as obese, and BMI ranging from 25 to 29.9 kg/m^2^ as overweight. There are 1.9 billion obese adults worldwide (25). Higher BMI is a risk factor of insulin resistance. Increased free fatty acids cause peripheral insulin resistance, as well as hampers the secretion of insulin by beta cells of the pancreas, resulting in elevated blood glucose levels (26, 27). In the research conducted by Holman et al, results showed that lower BMI at diagnosis might be related with rate of higher remission. Compared with the younger generation, older people achieved higher remission incidence due to their lower baseline BMI in average (28).Losing weight is encouraged in order to achieve better blood glucose control (12). Although in many clinical guidelines, low NaCl intake for type 2 diabetes patients is widely recommended, most patients tend to not act accordingly (29). Zhao et al. found that high NaCl intake could activate PPARδ in adipose tissues. Renal SGLT2 could be inhibited by the overexpression of adiponectin, thus the reabsorption of glucose is hindered, resulting in glycosuria (30). Alkaline phosphatase is found in multiple organs and it participates in various physiological processes. Wan et al’s research found that in patients who suffer from T2D, AKP levels and HbA1c levels were positively correlated, increased AKP levels could aggravate insulin resistance, and high serum AKP level could exasperate hyperglycemia (31). Although empirically, pulse seems to be not directly related to the level of blood glucose, type 2 diabetes could cause disturbances in the autonomic system. Inamdar’s research found that in comparison with normal people, the pulse rate of diabetic patients was higher. Pulse served as an independent risk factor for blood glucose disturbances, since it could be an barometer of the function of the autonomic nervous system (32). There is positive correlation between activated sympathetic nervous system and insulin resistance, which could affect patients’ blood glucose levels (33).

It is feasible to predict blood glucose levels *via* machine learning methods. The process is based on performance prediction indexes and the efficiency of this procedure is assessed using clinically derived datasets. The algorithms are helpful in case of limited data availability as the results of the models demonstrate the accuracy of predicting ability of the training and testing datasets. The physiological process of blood glucose regulation is complicated with various parameters and mechanisms. Herein, we predicted patients’ blood glucose levels using five machine learning algorithms (random forest regression, k-nearest neighbors, and logistic regression, support vector machine and XGBoost). To measure classification performance, we used different metrics in this study, such as accuracy, recall, precision, F1-score, and ROC curve. The binary confusion matrix was applied. “True Positive” represents patients with uncontrolled blood glucose were actually classified as such, “True Negative” represents patients with controlled blood glucose were labelled accordingly. “False Positive” signifies that patients who controlled their blood glucose were classified as uncontrolled, correspondingly “False Negative” implies that patients with uncontrolled blood glucose were regarded as the opposite. Evaluation indicators were calculated based on these. Accuracy depicts the performance in general. F1 score was calculated based on the value of precision and recall. In classifiers, “True Positive Rate” and “False Positive Rate” were represented in ROC curves. A high value of AUC delineates a high performance of the model (34). We assessed the outputs of the five machine learning models and compared them for the precise evaluation and prediction of blood glucose levels through clinical and numerical performance measures, and the results found that XGBoost is the ideal choice to assist better decision-making in the treatment of diabetic patients. Algorithms employed for predictive modelling tend to learn patterns from the provided data while ignoring irrelevant information from the data set at the same time. It has been observed that complicated and flexible machine learning strategies perform very well on training data but show poor results when applied to new data sets as a result of over-fitting. Applications using machine learning algorithms should avoid overfitting by techniques including feature selection and systemic cross-validation. Successful application will help users generate customized patient models for effective diabetes management (35).

The application of this research will allow diabetic patients to estimate their blood glucose levels with minimal intervention. There is limitation of this research: the sample size was relatively small, hence the parameters identified were limited. The result of the testing dataset was not as good as the training dataset, which could also be attributed to the small sample size. In the subsequent studies, we plan to recruit more patients for evaluation of the machine learning models in order to achieve performance improvement. There’s population bias in this study, since the participants were all over the age of 60 and the results might encounter extrapolation issues. We’d like to cover more age groups in the future.

## 5 Conclusion

We conducted blood glucose prediction using five machine learning algorithms (KNN, Logistic regression, Random Forest, Support Vector Machine, and XGBoost). Our research found that the XGboost algorithm performs better than other models with the most accuracy in prediction. Clinicians could use this algorithm to classify those with high risk of glycemic control failure, pay more attention to these patients and guide their lifestyle and adjust medications. XGBoost has the potential to assist in the effective management of diabetic patients in the future practice.

## Data availability statement

The original contributions presented in the study are included in the article/supplementary materials. Further inquiries can be directed to the corresponding authors.

## Ethics statement

The studies involving human participants were reviewed and approved by Medical Ethics Committee of the Chinese PLA General Hospital gave approval to this research (No. S2015-038-01). The patients/participants provided their written informed consent to participate in this study.

## Author contributions

HL and SY designed this study. NL and JL collected data. DK, JL and RC conducted analysis. XF and YW drafted the manuscript. All authors contributed to the article and approved the submitted version.
